# Polypoid Müllerianosis Mimicking Primary Gynecologic Malignancy

**DOI:** 10.1097/og9.0000000000000103

**Published:** 2025-07-31

**Authors:** Kathryn Quillen, H. James Williams, Krista S. Pfaendler

**Affiliations:** Department of Obstetrics, Gynecology, and Reproductive Sciences, and the Department of Pathology, Anatomy & Laboratory Medicine, West Virginia University, Morgantown, West Virginia.

## Abstract

**BACKGROUND:**

*Müllerianosis* is the ectopic presence of at least two distinct müllerian-derived tissues in extrauterine sites. This case report explores its origin, diagnosis, and treatment strategies.

**CASE:**

We report a biopsy-proven case of polypoid müllerianosis in the left distal ureter and parametrium, causing severe hydroureteronephrosis. Initial imaging raised concern for malignancy; however, histopathologic examination confirmed a benign lesion—polypoid müllerianosis. The patient underwent a modified radical hysterectomy with partial ureteral resection and reimplantation to relieve the obstruction and resect the affected segment en bloc with the primary gynecologic mass.

**CONCLUSION:**

This case highlights the importance of a multidisciplinary approach in diagnosing rare conditions such as polypoid müllerianosis. Collaborative efforts between gynecologists, urologists, and pathologists are essential for accurate diagnosis and optimal treatment planning.

## Abstract 2

Clinical mimicry of biopsy-proven müllerianosis resembling a primary gynecologic neoplasm highlights the importance of interprofessional collaboration to accurately obtain a diagnosis and provide optimal care.

**Teaching Points**

1. There are two main theories regarding the origin of müllerianosis: the implantation theory (implantation of müllerian tissues during pelvic surgery or cesarean delivery) and the metaplastic theory (differentiation of müllerian epithelium into different components within ectopic sites). These theories aid in the understanding of the pathophysiology of müllerianosis.
2. Distinguishing müllerianosis from primary gynecologic malignancies based on clinical presentation alone is a diagnostic challenge. Detailed histopathologic examination is essential for confirming the diagnosis. A defining feature of müllerianosis is the presence of at least two distinct müllerian derived tissue types—endometrial, endocervical, or tubal epithelium. Recognition of this characteristic admixture helps distinguish müllerianosis from malignant processes to prevent misdiagnosis and unnecessary aggressive treatment.
3. Radiologic imaging plays a vital role in the initial evaluation of müllerianosis. Imaging modalities such as ultrasonograpy, computed tomography, and magnetic resonance imaging can aid in visualizing the extent of the lesion and its relationship with surrounding structures and guide surgical planning.

*Polypoid müllerianosis* is the ectopic presence of at least two müllerian-derived tissues (endosalpinx, endometrium, or endocervix) in locations outside of the uterus.^1^ Unlike classic endometriosis, müllerianosis manifests within the organ rather than on the outer surface.^2^ Two theories of origin have been proposed: the implantation theory, in which müllerian tissues implant inside an ectopic site during pelvic surgery or cesarean delivery, and the metaplastic theory, in which müllerian epithelium differentiates into endometrial, endocervical, and tubal components within an ectopic site.^3–5^ Although müllerianosis primarily affects the urinary bladder, instances have been documented in abdominal scars, pelvic lymph nodes, and throughout the urinary tract.^6^ We present a case of biopsy-proven müllerianosis in a patient with obstructive uropathy secondary to a soft tissue mass involving the cervix, proximal vagina, parametrium, and left ureter, requiring modified radical hysterectomy with partial ureteral resection and reimplantation.

## CASE

**Fig. 1.**
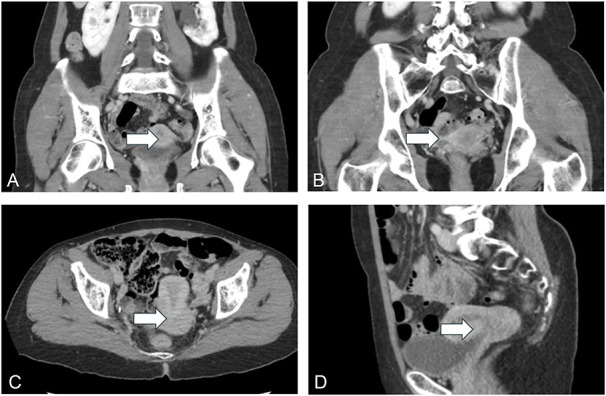
**A.** Narrowing of the left distal ureter (*arrow*) at the level of the soft tissue lesion. **B.** Circumferential soft tissue lesion (*arrow*) involving the distal left ureter with associated severe hydroureteronephrosis. **C.** Axial view of heterogeneous mass (*arrow*) at the level of the cervix. **D.** Sagittal view of heterogeneous mass (*arrow*) at the level of the cervix.

A 57-year-old nulliparous, postmenopausal patient presented with a month-long history of abdominal discomfort, nausea, pelvic pressure, urinary urgency and frequency, and left flank pain. Initial imaging revealed stricturing of the left distal ureter with severe hydroureteronephrosis (Fig. 1A). On further imaging, an abnormal soft tissue lesion surrounding the point of stricture (Fig. 1B) and a 4-cm cervical mass (Fig. 1C and D) were identified, raising concern for malignancy.

*Quillen. Polypoid Müllerianosis. O&G Open 2025*.

**Fig. 2.**
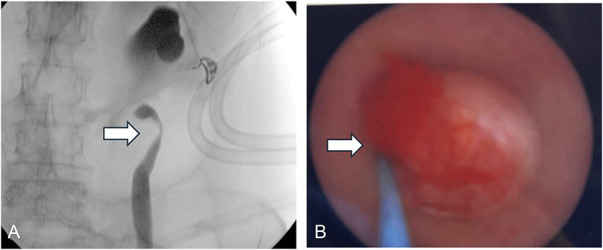
**A.** Intraoperative retrograde pyelography with severe hydroureteronephrosis to the level of narrowing in the distal ureter and significant J-hooking of the proximal ureter (*arrow*). **B.** Polypoid mass (*arrow*) within the left distal ureter consistent with filling defect on retrograde pyelogram.

The patient underwent surgical intervention to obtain biopsies of the suspected cervical mass and to alleviate the hydroureteronephrosis by insertion of a ureteral stent. During pelvic examination, a polypoid lesion of the left posterior cervicovaginal fornix was noted and biopsied, but no cervical mass was identified on visual inspection or palpation. Due to the high suspicion of cervical pathology, a random cervical biopsy was performed. Intraoperative retrograde pyelography revealed pronounced left hydroureteronephrosis to the level of the ureter's narrowing point, accompanied by significant J-hooking of the proximal ureter, requiring the placement of a ureteral stent (Fig. 2A). Vaginal biopsy demonstrated benign polypoid tissue, and cervical biopsy was consistent with a low-grade squamous lesion.

*Quillen. Polypoid Müllerianosis. O&G Open 2025*.

**Fig. 3.**
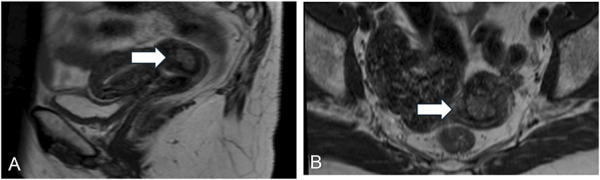
**A. **Sagittal view of lobulated mass (*arrow*) of the cervix inseparable from the left parametrium and left distal ureter, highly suspicious for malignancy. **B. **Axial view of lobulated mass (*arrow*) inseparable from the left parametrium and left ureter with internal solid-appearing enhancement.

A comprehensive review of the patient's pathology and imaging by a multidisciplinary tumor board prompted further investigation given the absence of a cervical mass on pelvic examination despite radiographic evidence and the benign pathologic findings from the vaginal biopsy. Pelvic magnetic resonance imaging was performed to further characterize the soft tissue mass; this revealed a cervical mass inseparable from the vagina, left parametrium, and left distal ureter causing hydroureteronephrosis (Fig. 3A and B), with an associated pelvic soft tissue mass suggestive of an atypical lymph node. Because a biopsy of the mass could not be obtained on speculum examination of the cervix, cystourethroscopy was performed with the aim of obtaining a transurethral biopsy. A polypoid mass consistent with the previously noted filling defect in the left distal ureter was visualized and biopsied (Fig. 2B). Histopathologic analysis of the left ureteral mass revealed a mix of endocervical and tubal epithelium, consistent with the characteristic features of polypoid müllerianosis.

*Quillen. Polypoid Müllerianosis. O&G Open 2025*.

**Fig. 4.**
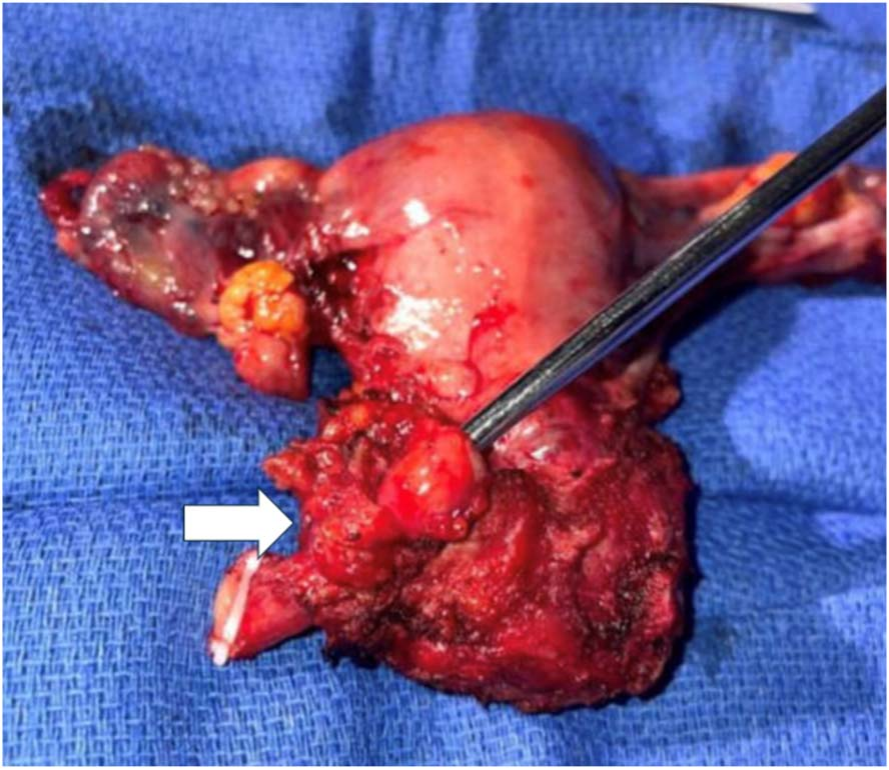
Uterus, bilateral fallopian tubes and ovaries, cervix, left parametrium, and 3-cm segment of distal left ureter enveloped by biopsy-proven müllerianosis mass (*arrow*).

The patient underwent a combined surgical procedure involving both gynecologic and urologic oncologists. Intraoperatively, a 3-cm segment of the left distal ureter was noted to be circumferentially enveloped in the parametrial mass (Fig. 4A). Consequently, the decision was made to proceed with planned robotic-assisted modified radical hysterectomy with en bloc resection of the involved 3-cm segment of distal ureter, with ureteroneocystostomy, psoas hitch, and stent placement.

*Quillen. Polypoid Müllerianosis. O&G Open 2025*.

**Fig. 5.**
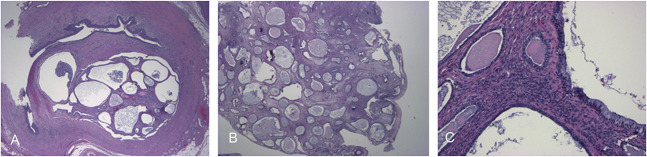
**A. **Hematoxylin and eosin (H&E) stain at 2× magnification demonstrating polypoid müllerianosis within the lumen of the left ureter causing compression and obstruction, with resulting hydronephrosis.** B.** H&E stain at 2× magnification demonstrating polypoid müllerianosis within the posterior cervicovaginal fornix, with admixed cysts demonstrating endosalpinx, endocervical, and endometrial epithelial linings. **C.** H&E stain at 20× magnification demonstrating müllerianosis found within the endocervical stroma, with the admixture of endosalpinx, endocervix, and endometrium.

Final pathology at the various sites, including the left ureter lumen polypoid mass (Fig. 5A), vaginal polyp (Fig. 5B), cervical stroma (Fig. 5C), parametrium, and uterus, demonstrated similar findings. These polypoid masses were composed of a cystic admixture of benign endosalpinx, endocervix, and endometrial glandular and stromal tissues. These findings supported the diagnosis of müllerianosis. During the patient's postoperative assessment, symptoms had improved and follow-up renal ultrasonography demonstrated a notable decrease in the left hydroureteronephrosis.

*Quillen. Polypoid Müllerianosis. O&G Open 2025*.

## DISCUSSION

Müllerianosis most often presents as a polypoid mass of the dome or posterior wall of the bladder.^6^ Although it predominantly affects the urinary bladder, instances have been documented in various anatomical sites including the ovary, vagina, pelvic lymph nodes, and regions of the urinary tract such as the ureter.^6–9^ The exact etiology of müllerianosis is still unknown, but two main theories of origin have been suggested. Young and Clement^4^ propose the implantation theory, in which müllerian tissue implants in a site outside of the uterus during pelvic surgery or cesarean delivery. This theory supports the pathogenesis of müllerianosis in more than half of the reported cases in the medical literature where the patient has previously undergone a cesarean delivery. This theory, however, does not explain the development of müllerianosis in patients without pelvic surgery, such as in the present case. Donné et al^5^ suggested the metaplastic theory, in which müllerian epithelium differentiates into endometrial, endocervical, and tubal components. This theory is supported by the embryologic origin of the müllerian system. During embryogenesis, the müllerian ducts fuse, forming the uterus, cervix, and proximal one-third of the vagina. This is known as the primary müllerian system. The secondary müllerian system refers to the peritoneal mesothelium and mesenchyme of the pelvis.^10^ Branca and Barresi^11^ suggest that this layer may have retained the potential to differentiate into normal or neoplastic tubal, endometrial, and endocervical epithelium. The present case could be explained by the metaplastic theory: the epithelial cells of the mesothelium in the mesosalpinx could differentiate into the aforementioned tissue types, leading to a benign polypoid lesion, or polypoid müllerianosis, as in our patient.^12^

There is currently no gold standard for treating müllerianosis as there is for classic endometriosis. Documented strategies have included medical and surgical approaches; however, there are minimal data on the management of this disease.^11^ Pharmacologic treatment could involve combined oral contraceptive pills, progestins, or gonadotropin-releasing hormone agonists.^3^ The rationale behind this treatment strategy is the responsiveness of müllerian glands and stroma to hormones due to their expression of estrogen and progesterone receptors, as in endometriosis.^11^ These pharmacotherapies down-regulate or suppress the hypothalamic-pituitary-axis, leading to suppression of hormones and, thus, possible involution of these lesions. This approach likely would be favored in cases in which surgical intervention is contraindicated, such as in medically complex individuals or with lesions in suboptimal locations.^3,11^

In one case, a patient was treated with a gonadotropin-releasing hormone analogue; after 2 years, the patient was found to be asymptomatic, with a smaller yet persistent lesion on follow-up.^13^ Surgical intervention often is preferred in symptomatic cases involving the bladder, where resection could be performed with ease, or in cases with hydronephrosis or ureteral obstruction, as in the present case, to prevent further renal impairment.^3,11^

There is more literature on the approach to surgical treatment of bladder lesions, because bladder involvement is more common than ureteral involvement. Case reports have mentioned transurethral resection and both open and laparoscopic partial cystectomy.^11,14,15^ A thorough literature review revealed three documented cases of müllerianosis involving the ureter and one documented case involving the mesosalpinx.^9,16–18^ Surgical resection was chosen as the treatment modality for all three ureteral lesions. In one case, segmental ureteral resection with ureteroureteral anastomosis was performed to address the affected portion of the ureter.^9^

Our case differs because the lesion involved more than just the ureter. For that reason, the uterus, cervix, parametrium, upper vagina, ovaries, fallopian tubes, broad ligament, and the affected left distal ureter were removed. We performed a modified radical hysterectomy on the involved side and simple hysterectomy on the contralateral side, because there was preservation of supporting structures and parametrium on the contralateral side due to unilateral involvement of the parametrium and distal ureter.^19^ The hydroureteronephrosis notably improved in the weeks after surgery, and the patient's urinary urgency and frequency and flank pain completely resolved.

This exceptionally rare case of biopsy-proven polypoid müllerianosis is of importance because it highlights the diagnostic challenges a clinician may face when trying to distinguish müllerianosis from other primary gynecologic neoplasms. Diagnosis and treatment involves the collaboration and coordination of care by gynecologic oncologists, urologic oncologists, and pathologists to guide and implement the appropriate approach to treatment that achieves optimal outcomes and minimizes patient morbidity.
